# Determining Fuzzy Membership for Sentiment Classification: A Three-Layer Sentiment Propagation Model

**DOI:** 10.1371/journal.pone.0165560

**Published:** 2016-11-15

**Authors:** Chuanjun Zhao, Suge Wang, Deyu Li

**Affiliations:** 1 School of Computer and Information Technology, Shanxi University, Taiyuan, 030006, Shanxi, China; 2 Key Laboratory of Computational Intelligence and Chinese Information Processing of Ministry of Education, Taiyuan, 030006, Shanxi, China; Tianjin University, CHINA

## Abstract

Enormous quantities of review documents exist in forums, blogs, twitter accounts, and shopping web sites. Analysis of the sentiment information hidden in these review documents is very useful for consumers and manufacturers. The sentiment orientation and sentiment intensity of a review can be described in more detail by using a sentiment score than by using bipolar sentiment polarity. Existing methods for calculating review sentiment scores frequently use a sentiment lexicon or the locations of features in a sentence, a paragraph, and a document. In order to achieve more accurate sentiment scores of review documents, a three-layer sentiment propagation model (TLSPM) is proposed that uses three kinds of interrelations, those among documents, topics, and words. First, we use nine relationship pairwise matrices between documents, topics, and words. In TLSPM, we suppose that sentiment neighbors tend to have the same sentiment polarity and similar sentiment intensity in the sentiment propagation network. Then, we implement the sentiment propagation processes among the documents, topics, and words in turn. Finally, we can obtain the steady sentiment scores of documents by a continuous iteration process. Intuition might suggest that documents with strong sentiment intensity make larger contributions to classification than those with weak sentiment intensity. Therefore, we use the fuzzy membership of documents obtained by TLSPM as the weight of the text to train a fuzzy support vector machine model (FSVM). As compared with a support vector machine (SVM) and four other fuzzy membership determination methods, the results show that FSVM trained with TLSPM can enhance the effectiveness of sentiment classification. In addition, FSVM trained with TLSPM can reduce the mean square error (MSE) on seven sentiment rating prediction data sets.

## Introduction

Following the popularization of forums, blogs, and online shopping websites, amount of user-generated reviews are growing explosively [1]. Techniques for extracting, arranging, and drawing conclusions from these multitudinous reviews, and in particular, for classifying them according to their sentiment orientation and sentiment intensity are receiving an increasing amount of interests from researchers and manufacturers [2]. In general, customers frequently utilize the Internet to search for related comments about an item before purchasing. Meanwhile, manufacturers want to obtain the customers’ advice so as to improve the product design as well. Thus, the classification of this information according to sentiment tendency is very convenient for both manufacturers and customers. Sentiment classification is aimed to recognize sentiment information hidden in the texts automatically, for example, opinions, emotions, and standpoints [3]. In addition, the applications of sentiment classification are also extensive, such as text filtering, e-business, and public opinion prediction [4].

As compared with traditional classification tasks, sentiment classification is relatively challenging. A deep semantic analysis of the documents is required to judge the sentiment orientation [5, 6]. Supervised machine learning models, such as, support vector machine (SVM), decision tree, and bayesian classification, have been applied to the text sentiment classification task. Among those models, SVM has achieved effective results [7]. However, SVM assigns equal weight to all samples, while different samples affect or contribute to the classification surface very differently [8, 9]. Fuzzy support vector machine (FSVM) introduces fuzzy membership to the SVM. Each sample is assigned a value of fuzzy membership. The samples which are noisy data or make small contributions to the classification have a lower weight, and the samples that make greater contributions to the classification have a higher weight. Using this strategy, FSVM gives different fuzzy memberships to samples contributing a different amount to classification [10, 11]. Comparing with SVM, FSVM can improve the classification accuracy and reduce adverse effects from the noisy data.

Clearly, sentiment scores can describe the sentiment orientation and sentiment intensity of documents in great detail. It is hard for human beings to estimate the accurate sentiment score of a specified document and the results are also unreliable [12]. Therefore, techniques for capturing sentiment scores automatically are very important. In order to obtain the sentiment score of review documents, researchers have adopted a sentiment lexicon to count the positive and negative words and their sentiment intensity. In addition to the sentiment words and sentiment lexicon, researchers have also used the distance to the class centroid for measuring the fuzzy membership [13]. FSVM has been proved to be effective in theory and applications for classification task. In sentiment classification, we should construct the membership function according to the characteristics of data set and data features. In FSVM, the key is to determine the appropriate fuzzy membership of samples. Fuzzy sentiment membership should reflect the contribution degree of a document to sentiment classification. Generally, we think that strong sentiment intensity of positive or negative documents make large contributions to sentiment classification, while weak sentiment intensity samples are unimportant. Therefore the stronger is the sentiment intensity of documents, the bigger is degree of membership to the sentiment labels. To get more accurate sentiment classification results, we use the absolute value of sentiment score as the fuzzy membership to train the FSVM.

To determine the fuzzy sentiment membership of documents, we adopt a three-layer sentiment propagation model (TLSPM). In this context, the so-called three layers refer to documents, topics, and words. First, we construct nine relationship pairwise matrices between documents, topics, and words. The sentiment score of documents, topics, and words are determined by their sentiment neighbors. Then we obtain a steady sentiment score through continuous iterations. In order to achieve better sentiment classification results, we give higher weights to training samples having a strong sentiment intensity of positive or negative polarity, and lower weights to those having weak sentiment intensity. By using these weighted training samples, a text sentiment classifier of an FSVM can be obtained. Fifteen frequently used real-world sentiment data sets, including eight two-class data sets and seven multi-level data sets, were selected to evaluate the effectiveness of the proposed method. As compared with SVM and four other fuzzy membership determination methods, the experimental results show that FSVM trained with TLSPM can increase the accuracy of sentiment classification. In addition, FSVM trained with TLSPM can also reduce the mean square error (MSE) on seven sentiment rating prediction data sets.

## Related work

In this section, we briefly review the existing methods for two-class sentiment classification, sentiment rating prediction methods, and application of the topic model to sentiment classification.

### Two-class sentiment classification

Traditional text sentiment classification in general divides the reviews into positive or negative categories according to their sentiment orientation [14]. Current methods for two-class sentiment classification can be roughly divided into three approach categories: lexicon-based, semi-supervised, and supervised machine learning [15, 16].

#### Lexicon-based approach

Sentiment lexicons are widely used in the fine-grained sentiment analysis of reviews. The lexicon-based approach calculates the orientation of a document from the sentiment orientation of words or phrases in the document. Turney [17] proposed a simple unsupervised learning algorithm to predict the sentiment orientation using the average sentiment orientation of the phrases in the review. They first identified phrases that contained adjectives or adverbs using the part-of-speech tagger. In their method, the sentiment orientation of a phrase is calculated as the mutual information of the given phrase and the word “excellent”minus the mutual information of the given phrase and the word “poor”. If the average sentiment orientation of all its phrases is positive, the review is considered positive, and if negative, the review is considered negative. Ohana and Tierney [18] applied the SentiWordNet lexicon to the problem of automatic sentiment classification of film reviews. They determined sentiment orientation by counting positive and negative term sentiment scores. On this basis, they used machine learning methods to classify the reviews and found the relevant sentiment features using SentiWordNet. Through a comparative experiment, they found that the feature set approach was better than the sentiment term counting approach. Kanayama and Nasukawa [19] first detected polar clauses that conveyed positive or negative aspects, after which they built a sentiment lexicon that comprised polar atoms through an unsupervised method. The polar atoms were defined as the minimum syntactic elements that express sentiment. They used context coherency to obtain candidate polar atoms. They needed only untagged domain corpora and an initial lexicon to select the appropriate polar atoms from among candidates.

#### Semi-supervised approach

Sometimes, the labeled training data for sentiment classification are precious and scarce, while abundant unlabeled reviews are easier to get. By designing strategies or techniques, semi-supervised methods combine a certain amount of unlabeled data with the labeled data in the learning process. Wan [20] focused on the problem of cross-lingual sentiment classification, and leveraged an available English corpus for Chinese sentiment classification by using the English corpus as training data. They first used machine translation methods to reduce the gap between Chinese and English. In their method, English features and Chinese features are considered two independent views of the classification problem. They proposed a co-training approach to utilize unlabeled Chinese data. Li et al. [21] adopted two views, personal and impersonal, and employed them in both supervised and semi-supervised sentiment classification systematically. In this method, personal views consist of those sentences that directly express a speaker’s feelings and preference for a target object, while impersonal views focus on statements about a target object for evaluation. Based on this, an ensemble method and a co-training algorithm are explored to employ the two views in supervised and semi-supervised sentiment classification, respectively. Yu et al. [16] proposed a semi-supervised approach to solve the imbalance between the subjective and objective classes in the twitter sentiment task. The emotion sentiments automatically was extracted from the tweets, and the required training data set was selected in an automatic manner. With more and more social media users sharing their opinions with additional images and videos, You et al. [22] presented a cross-modality consistent regression (CCR) model, which was able to utilize both the state-of-the-art visual and textual sentiment analysis techniques.

#### Supervised machine learning approach

Supervised sentiment classification methods employ mainly supervised machine learning methods, such as decision tree, naive bayes, SVM, and neural networks [23]. Based on the words that convey sentiment, a new feature selection method based on matrix factorization was proposed by Liang et al. [14] to identify the words with strong inter-sentiment distinguish-ability and intra-sentiment similarity. Ye et al. [24] compared three supervised machine learning methods, naive bayes, SVM, and the character-based N-gram model, for sentiment classification of the reviews of travel blogs for seven popular travel destinations. After experimental verification, they found that the SVM and N-gram approaches performed better than the naive bayes method. The three machine learning approaches reached at least 0.8 when the training set was sufficiently large. Facing with encoding the intrinsic relations between sentences in the semantic meaning of document, Tang et al. [1] presented a neural network approach to learn continuous document representation for sentiment classification. They reported that gated recurrent neural network outperformed traditional recurrent neural network. Aiming at target-dependent Twitter sentiment classification task, Vo et al. [15] explored a rich set of neural pooling functions for automatic feature extraction, drawing theoretical correlations behind these functions.

### Sentiment rating prediction

It is worth noting that most studies of sentiment classification in general divide the reviews into positive and negative categories. This is because two-class sentiment classification is relatively simple, being concerned only with the polarity of the comments and not considering the sentiment intensity of reviews. As compared with two-class sentiment classification, sentiment rating prediction is a challenging task. Not only does it judge the sentiment orientation, but also it classifies the reviews into more detailed categories [25, 26].

Pang and Lee [27] applied a meta-algorithm based on a metric labeling formulation of the problem, which altered a given n-ary classifier’s output in an explicit attempt to ensure that similar items were assigned similar labels. They showed that the meta-algorithm could provide significant improvements over both multi-class and regression versions of SVM when a novel similarity measure appropriate to the problem was employed.

Qu et al. [28] captured the sentiment polarity and intensity of N-grams by introducing a novel kind of bag-of-opinions representation. In their method, each opinion is composed of a root word, a set of modifier words from the same sentence, and one or more negation words. For example, in the opinion “not very helpful”, “helpful”is the root word, “very”is the modifier word, and “not”is the negation word. On this basis, they obtained the sentiment score of each opinion using a constrained ridge regression method over a large number of domain-independent reviews. The ratings of test reviews were determined using the sentiment score of all the opinions in the review and a domain-dependent unigram model. As compared with the previous sentiment ratings prediction methods, its validation for books, movies, and music data sets showed the effectiveness of the bag-of-opinions model.

Long et al. [29] proposed a novel review selection approach for accurate feature rating estimation. They used a bayesian network classifier to predict the sentiment star for each topic in the reviews. In order to achieve better results, their approach selected only those reviews that were related to the topics by using the Kolmogorov complexity (KC) information measure. The rating estimation of the feature for these selected reviews using machine learning techniques provided more accurate results than that for other reviews. The average of these estimated feature ratings also better represented an accurate overall rating for the feature of the service, which provided feedback that helped other users to choose their satisfactory service.

Snyder and Barzilay [30] formulated the sentiment rating prediction task as a multiple aspect ranking problem, where the goal was to produce a set of numerical scores, one for each aspect. They presented an algorithm that jointly learned ranking models for individual aspects by modeling the dependencies between assigned ranks. This algorithm guided the prediction of individual rankers by analyzing meta-relations between opinions, such as agreement and contrast. They proved that an agreement-based joint model was more expressive than individual ranking models.

Wang et al. [31] proposed a new opinionated text data analysis called latent aspect rating analysis (LARA). To analyze the sentiment star of topical aspects, LARA started with some reviews with sentiment ratings, particular aspects in the reviews, and each reviewer’s ratings for a given aspect. To achieve this deeper and more detailed understanding of a review, they proposed a two-stage approach based on a novel latent rating regression model. First, they adopted a bootstrapping method to select the main aspects and segments of reviews. In the second stage, a new generation of the latent rating regression model (LRR) was trained to predict aspect ratings. An important assumption was that the overall rating was generated based on a weighted combination of the latent ratings over all the aspects. Their evaluation using a hotel data set showed the effectiveness of the latent rating regression model and the aspect ratings generation assumption.

### Application of the topic model to sentiment classification

The LDA model can detect topics that are implicit in the texts and achieved great success in the text mining field [32, 33]. In LDA, the generation process is defined as follows. (i) For each document, extract a topic from the topic distribution; (ii) extract a word from the topic to be able to get above the word corresponding to the distribution; and (iii) repeat the process until every word in the document has been traversed. The application of a topic model in sentiment analysis can improve the performance of sentiment analysis by mining the topics that are implicit in the texts and sentiment preferences for the topics.

Mei et al. [34] proposed a novel probabilistic model to capture the mixture of topics and sentiments simultaneously. They proposed a topic-sentiment mixture model (TSM). TSM first classified the words into two categories, where one category was irrelevant to the topics, and the other was related to the topics. Then, the second category was divided into positive, negative, and neutral sub-categories, and the probability distribution of words in each class was estimated by using an EM algorithm. Finally, particular topic life cycles and the relationship between topics and sentiment were extracted.

Li et al. [35] assumed that topics in texts were relevant to the sentiment and proposed the sentiment topic joint model (Sentiment-LDA model). Based on this, they found that sentiment was independent of the local context, and they proposed the Dependency-Sentiment-LDA model, in which the sentiment of the words in the text formed a Markov chain, and the sentiment of a word was independent of the previous word.

Lin et al. [36] proposed a novel probabilistic model framework called the joint sentiment-topic model(JST), and the re-parameterization JST model called the Reverse-JST model. Both methods were weakly supervised, and therefore, they could easily be adapted to other domains. Joint sentiment-topic models added the sentiment layer into the text layer and topic layer to form four models. The reverse-JST model was also a four-layer Bayesian model, but the sentiment generation process was independent of the topics, as compared with JST.

## Three-layer sentiment propagation model

To determine the fuzzy sentiment membership of documents, three-layer sentiment propagation model (TLSPM) makes full use of the relationships between document, topics, and words by using pre-existing tools, such as cosine distance, LDA, and fisher feature selection methods. An important advantage of TLSPM is to construct the sentiment propagation network and its matrix representation between documents, topics, and words. In the sentiment propagation process, the sentiment forming of a document, a topic, or a word is regarded as a propagation process on its carriers. At the same time, the sentiment scores of a document, a topic, and a word are determined by their sentiment neighbors in the sentiment network. The matrix representation of the sentiment network and three kinds of sentiment propagation process including toward document, toward topic, and toward word in the sentiment network are unified in sentiment propagation algorithm. In sentiment propagation algorithm, the sentiments of documents, topics, and words are imaged as a steady state of the sentiment network after the propagation process. After consecutive iterations of sentiment score sets of documents, topics, and words through sentiment neighbors propagation, we get the fuzzy membership set of documents and fuzzy training document set.

### Symbols and notions

*C*_1_ = {1, −1}: Two-class sentiment label set, where 1 represents a positive tendency and −1 represents a negative tendency.

*C*_2_ = {1, 2, 3, 4, 5}: Sentiment rating prediction label set, where 1 represents a strong negative tendency, 2 represents a negative tendency, 3 represents a neutral sentiment tendency, 4 represents a positive tendency, and 5 represents a strong positive tendency.

*D* = {*d*_1_, *d*_2_, ⋯, *d*_*N*_}: Document set. Each *d*_*i*_ in *D* has a sentiment label.

*T* = {*t*_1_, *t*_2_, ⋯, *t*_*l*_}(1 ≤ *h* ≤ *l*): Topic set. A topic *t*_*h*_ is a series of words.

*W* = {*w*_1_, *w*_2_, ⋯, *w*_*m*_}(1 ≤ *j* ≤ *m*): Word set. Each *w*_*j*_ in *W* has a sentiment label.

**Sentiment score**: Sentiment score measures the sentiment tendency and intensity of a document (*d*_*i*_), topic (*t*_*h*_), or word (*w*_*j*_). Their sentiment score are denoted by *score*(*d*_*i*_), *score*(*t*_*h*_), and *score*(*w*_*j*_), respectively.

**Fuzzy sentiment membership of a document**: The absolute value |*score*(*d*_*i*_)| of the sentiment score *score*(*d*_*i*_) is defined as the fuzzy sentiment membership of a document (*d*_*i*_).

**Fuzzy training document set**: The fuzzy training document set is defined as $D_{train}^{F}=\left\{\left(d_{1},y_{1},s_{1}\right),\left(d_{2},y_{2},s_{2}\right),\cdots ,\left(d_{n},y_{n},s_{n}\right)\right\}$, where *d*_*i*_ is a document, *y*_*i*_ is the sentiment label of *d*_*i*_, *s*_*i*_ is the fuzzy sentiment membership of *d*_*i*_, and (*d*_*i*_, *y*_*i*_, *s*_*i*_) is a fuzzy training sample. *S* = {*s*_1_, *s*_2_, ⋯, *s*_*n*_} is the fuzzy membership set.

**Fig 1:**
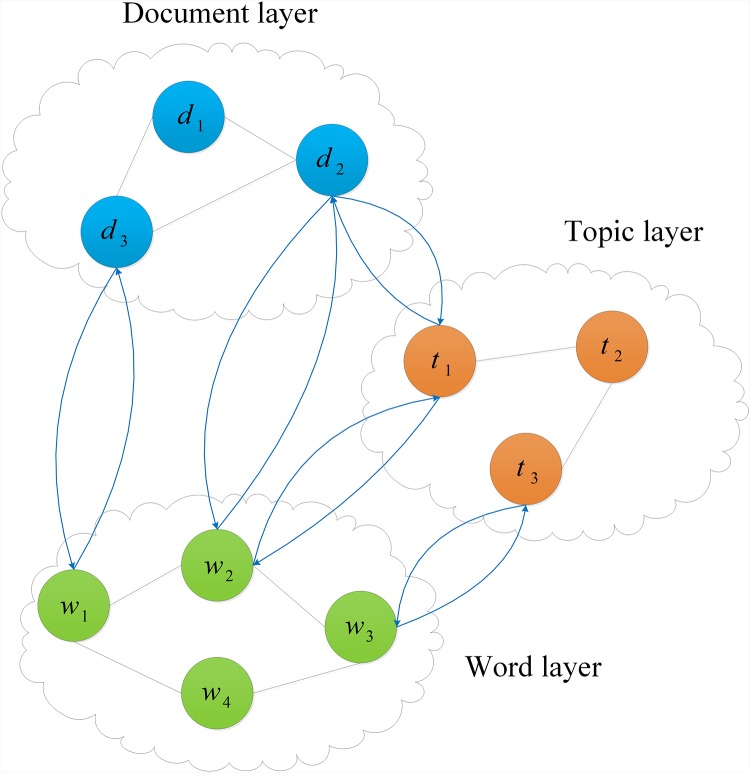
Sketch of the three-layer sentiment network.

**Three-layer sentiment network**: This network is a weighted directed graph, which is used to describe the relationships among documents, topics, and words. A document (*d*_*i*_) is composed of most relevant topics, meanwhile a topic (*t*_*h*_) is composed of most relevant words (*w*_*j*_). In the graph, if a kind of relation is symmetric, the corresponding bidirectional edges are then drawn as undirected lines. The weight of an edge expresses the relation intensity of both nodes linked by the edge. It should be noted that the sentiment information on the network is propagated together with the direction of the edges. A sketch of the structure of this network is shown in Fig 1.

**Neighbors in the sentiment network**: The neighbors of a node (document, topic, or word) in the sentiment network refer to other nodes that link toward the node. The larger the value of the relation intensity between two nodes in the sentiment network, the higher probability of their becoming sentiment neighbors. For example, in the Book domain, the words “good”and “excellent”are linked by a large value of relation intensity, and therefore, they are sentiment neighbors in the sentiment network.

### Matrix representation of the sentiment network

Let **G** = {(*D*_*train*_, *T*, *W*), *E*} be a sentiment network, where *E* is the set of weighted directional edges and each edge links two nodes from *D*, *T*, and *W*.

We know that any directed graph can be equivalently represented as its adjacent matrix. It is not difficult to see that the graph **G** can be divided into nine subgraphs with their adjacent matrices as $\tilde{\text{P}}$, $\tilde{\text{Q}}$, $\tilde{\text{M}}$, $\tilde{\text{N}}$, $\tilde{\text{U}}$, $\tilde{\text{V}}$, $\tilde{\text{G}}$, $\tilde{\text{H}}$, and $\tilde{\text{Z}}$, respectively.

$$\begin{array}{c} \text{G}=[\begin{array}{ccc} \tilde{\text{P}} & \tilde{\text{V}} & \tilde{\text{M}}\\ \tilde{\text{G}} & \tilde{\text{U}} & \tilde{\text{H}}\\ \tilde{\text{N}} & \tilde{\text{Z}} & \tilde{\text{Q}} \end{array}] \end{array}$$ (1)

Their definitions are given by constructing their adjacent matrices as below.

$\tilde{\text{P}}$: The adjacent matrix between documents. The weight of the edge related to documents *d*_*i*_ and *d*_*j*_ is defined as

$$\begin{array}{c} {\tilde{\text{P}}}_{ij}=\cos\left(d_{i},d_{j}\right)=\frac{d_{i}\cdot d_{j}}{\left|d_{i}|\times |d_{j}\right|} \end{array}$$ (2)

Here, *d*_*i*_ and *d*_*j*_ are also used to denote the vectors of documents *d*_*i*_ and *d*_*j*_, respectively.

$\tilde{\text{Q}}$: The adjacent matrix between words. The weight of the edge related to words *w*_*i*_ and *w*_*j*_ is defined as

$$\begin{array}{c} {\tilde{\text{Q}}}_{ij}=BNS\left(w_{i},w_{j}\right)=|F^{-1}\left(p\left(w_{i}|w_{j}\right)\right)-F^{-1}\left(p\left(w_{i}|\bar{w_{j}}\right)\right)| \end{array}$$ (3)

*F*^−1^ represents standard normal distribution of the accumulated anti-probability function, *p*(*w*_*i*_|*w*_*j*_) is the probability of *w*_*i*_ appearing when *w*_*j*_ appears in the same window, and $p(w_{i}|\bar{w_{j}})$ is the probability of *w*_*i*_ appearing when *w*_*j*_ does not appear. The bi-normal separation (BNS) method was proposed by Forman [37]. We set the window size as 10.

$\tilde{\text{M}}$: The adjacent matrix from words to documents. The weight of the edge from word *w*_*j*_ to document *d*_*i*_ is defined as

$$\begin{array}{c} {\tilde{\text{M}}}_{ij}=wei\left(d_{i},w_{j}\right)=\frac{tf_{w_{j}}\times idf_{w_{j}}}{\sum_{w\in d_{i}}tf_{w}\times idf_{w}} \end{array}$$ (4)

where *tf*_*w*_*j*__ is the word frequency of *w*_*j*_ in *d*_*i*_, *idf*_*w*_*j*__ is the inverse text frequency of *w*_*j*_, *idf*_*w*_*j*__ = 1 + log(*N*/*n*_*w*_), *N* is the total number of documents, and *n*_*w*_ is the number of documents that contain the word *w*_*j*_. ${\tilde{\text{M}}}_{ij}$ measures the contribution degree of word *w*_*j*_ to document *d*_*i*_.

$\tilde{\text{N}}$: The adjacent matrix from documents to words. The weight of the edge from document *d*_*j*_ to word *w*_*i*_ is defined as

$$\begin{array}{c} {\tilde{\text{N}}}_{ij}=wei\left(w_{i},d_{j}\right)=\frac{tf_{d_{j}}\times idf_{d_{j}}}{\sum_{d\in w_{i}}tf_{d}\times idf_{d}} \end{array}$$ (5)

where *tf*_*d*_*j*__ is the word frequencies of *d*_*j*_, *idf*_*d*_*j*__ is the inverse text frequency of *d*_*j*_, *idf*_*d*_*j*__ = 1 + log(*N*/*n*_*d*_), *N* is the total number of words, and *n*_*d*_ is the number of words that occur in the document *d*_*j*_. *d* ∈ *w*_*i*_ means that the document *d* contains the word *w*_*i*_.

$\tilde{\text{U}}$: The adjacent matrix between topics. The weight of the edge related to topics *t*_*i*_ and *t*_*j*_ is defined as

$$\begin{array}{c} {\tilde{\text{U}}}_{ij}=\cos\left(t_{i},t_{j}\right)=\frac{t_{i}\cdot t_{j}}{\left|t_{i}|\times |t_{j}\right|} \end{array}$$ (6)

Here, *t*_*i*_ and *t*_*j*_ are also used to denote the vectors of topics *t*_*i*_ and *t*_*j*_, respectively.

$\tilde{\text{V}}$: The adjacent matrix from topics to documents. The weight of the edge from topic *t*_*j*_ to document *d*_*i*_ is defined as

$$\begin{array}{c} {\tilde{\text{V}}}_{ij}=p\left(t_{j}|d_{i}\right) \end{array}$$ (7)

where *p*(*t*_*j*_|*d*_*i*_) is the weight of the topic *t*_*j*_ in document *d*_*i*_ in the LDA results, and measures the contribution degree of topic *t*_*j*_ to document *d*_*i*_.

$\tilde{\text{G}}$: The adjacent matrix from documents to topics. The weight of the edge from document *d*_*j*_ to topic *t*_*i*_ is defined as

$$\begin{array}{c} {\tilde{\text{G}}}_{ij}=p\left(d_{j}|t_{i}\right) \end{array}$$ (8)

where *p*(*d*_*j*_|*t*_*i*_) is the weight of the topic *t*_*i*_ in the document *d*_*j*_ in the LDA results, and measures the contribution degree of topic *d*_*j*_ to document *t*_*i*_.

$\tilde{\text{H}}$: The adjacent matrix from words to topics. The weight of the edge from word *w*_*j*_ to topic *t*_*i*_ is defined as

$$\begin{array}{c} {\tilde{\text{H}}}_{ij}=p\left(w_{j}|t_{i}\right) \end{array}$$ (9)

where *p*(*w*_*j*_|*t*_*i*_) is the weight of *w*_*j*_ in *t*_*i*_ in the LDA results, and measures the contribution degree of word *w*_*j*_ to topic *t*_*i*_.

$\tilde{\text{Z}}$: The adjacent matrix from topics to words. The weight of the edge from topic *t*_*j*_ to word *w*_*i*_ is defined as

$$\begin{array}{c} {\tilde{\text{Z}}}_{ij}=p\left(t_{j}|w_{i}\right) \end{array}$$ (10)

where *p*(*t*_*j*_|*w*_*i*_) is the weight of the word *w*_*i*_ in the topic *t*_*j*_ in the LDA results, and measures the contribution degree of word *t*_*j*_ to topic *w*_*i*_.

### Sentiment propagation process

Sentiment propagation in this paper refers neither to sentiment propagation among individuals in a social system nor to changes in the sentiment of individuals who already have certain sentiments in a system. In this paper, it means only the acquisition of a more precise sentiment depiction of review documents by using some known exact sentiment information about documents and words, middle layer nodes, i.e., topics hidden in documents, and the relationship among them on the sentiment network through information propagation.

**Fig 2:**
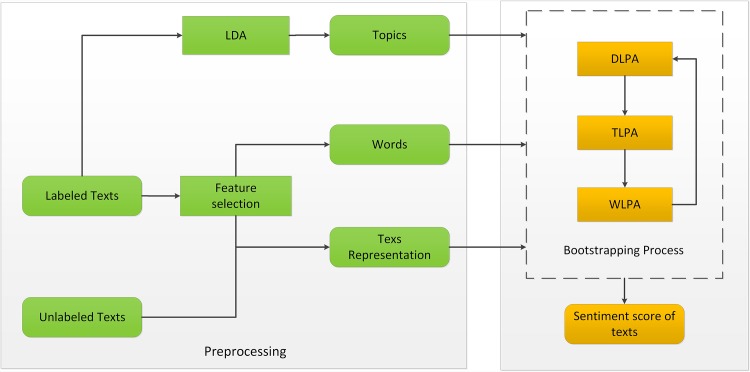
Sketch of three-layer sentiment propagation model.

It can be assumed that a review document is generated as follows. The author first selects one or more topics which interest him/her and then selects some of his/her favorite words to describe each topic using his/her sentiments. Therefore, in the aspect of generating review documents, the sentiment of a document determines the sentiments hidden in the document, and the sentiment of a topic determines the sentiments of the words that are related to the topic. Conversely, in the aspect of the composition of a document, the sentiment of a topic is determined by the words that are aggregated to the topic; the sentiments of topics and words determine the sentiment of a document. Therefore, we regard sentiment forming as a propagation process on its carriers, documents, topics, and words in the sentiment network. We image the sentiments of documents, topics, and words as a steady state of the sentiment network after the propagation process. Here, we assume that *score*(*d*_*i*_), *score*(*t*_*h*_), *score*(*w*_*j*_) are influenced by its sentiment neighbors. A flowchart of TLSPM can be seen in Fig 2.

We construct the sentiment score vector as

$$\begin{array}{c} score\left(D\right)={\left[score\left(d_{1}\right),score\left(d_{2}\right),\cdots ,score\left(d_{n}\right)\right]}^{T} \end{array}$$ (11)

$$\begin{array}{c} score\left(T\right)={\left[score\left(t_{1}\right),score\left(t_{2}\right),\cdots ,score\left(t_{l}\right)\right]}^{T} \end{array}$$ (12)

$$\begin{array}{c} score\left(W\right)={\left[score\left(w_{1}\right),score\left(w_{2}\right),\cdots ,score\left(w_{m}\right)\right]}^{T} \end{array}$$ (13)

We next design three kinds of sentiment propagation process in the sentiment network. In the following propagation formulas, *α*, *β*, and *γ* are the document weight, topic weight, and word weight, respectively, and are restrained by *α* + *β* + *γ* = 1.

Toward document (**ToD**):

$$\begin{array}{ccc} score(D) & = & \alpha \times \tilde{\text{P}}\times score\left(D\right)+\beta \times \tilde{\text{V}}\times score\left(T\right)\\ & & +\gamma \times \tilde{\text{M}}\times score\left(W\right) \end{array}$$ (14)

Toward topic (**ToT**):

$$\begin{array}{ccc} score(T) & = & \alpha \times \tilde{\text{G}}\times score\left(D\right)+\beta \times \tilde{\text{U}}\times score\left(T\right)\\ & & +\gamma \times \tilde{\text{H}}\times score\left(W\right) \end{array}$$ (15)

Toward word (**ToW**):

$$\begin{array}{ccc} score(W) & = & \alpha \times \tilde{\text{N}}\times score\left(D\right)+\beta \times \tilde{\text{Z}}\times score\left(T\right)\\ & & +\gamma \times \tilde{\text{Q}}\times score\left(W\right) \end{array}$$ (16)

**Remark 1**. The number of sentiment neighbors *k*.

According to the label propagation algorithm (LPA), which was proposed by Liu and Murata [38], *score*(*d*_*i*_), *score*(*t*_*h*_), and *score*(*w*_*j*_) in the propagation graph **G** are determined by its sentiment neighbors in the sentiment network. We use its neighbors to refer to other nodes that link toward the node.

At the initialization of the adjacent matrices stage, we prune the propagation graph **G** and keep *k* neighbors of each node. We keep *k* major values of each row in $\tilde{\text{P}}$, $\tilde{\text{Q}}$, $\tilde{\text{M}}$, $\tilde{\text{N}}$, $\tilde{\text{U}}$, $\tilde{\text{V}}$, $\tilde{\text{G}}$, $\tilde{\text{H}}$, and $\tilde{\text{Z}}$, assign 0 to the others, and normalize each row. In the process of sentiment propagation, we choose only the nearest *k* neighbors to determine *score*(*d*_*i*_), *score*(*t*_*h*_), or *score*(*w*_*j*_). At each step of the sentiment propagation, we update the sentiment score of each node by using adjacent nodes. The greater the similarity of the adjacent nodes, the stronger is the influence weight of adjacent nodes.

**Remark 2**. Initialization of adjacency matrices.

**Fig 3:**
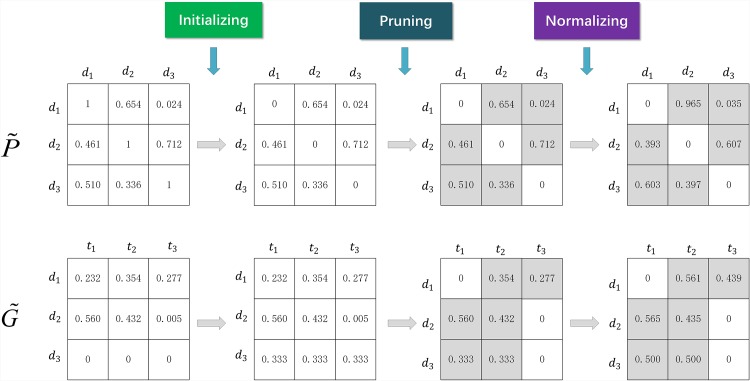
Two examples of adjacency matrices initialization.

For guaranteeing the convergence of the sentiment propagation algorithm, we initialize the adjacency matrices $\tilde{\text{P}}$, $\tilde{\text{Q}}$, $\tilde{\text{U}}$, $\tilde{\text{M}}$, $\tilde{\text{N}}$, $\tilde{\text{V}}$, $\tilde{\text{G}}$, $\tilde{\text{H}}$, and $\tilde{\text{Z}}$ as follows. Two examples of adjacency matrices initialization can be seen in Fig 3.

For $\tilde{\text{P}}$, $\tilde{\text{Q}}$, and $\tilde{\text{U}}$, we first set ${\tilde{\text{P}}}_{ii}=0$, ${\tilde{\text{Q}}}_{ii}=0$, and ${\tilde{\text{U}}}_{ii}=0$, if any row is 0, we assign 1/*k* to each element in the corresponding row. Then we keep the *k* major value of each row, and assign 0 to the others. Finally, we normalize each row of the matrices $\tilde{\text{P}}$, $\tilde{\text{Q}}$, and $\tilde{\text{U}}$.

For $\tilde{\text{M}}$, $\tilde{\text{N}}$, $\tilde{\text{V}}$, $\tilde{\text{G}}$, $\tilde{\text{H}}$, and $\tilde{\text{Z}}$, if any row is 0, we assign 1/*k* to each element in the corresponding row. Then we keep the *k* major values of each row, assign 0 to the others, and normalize each of their rows.

**Remark 3**. Initialization and normalization of sentiment score.

For the two-class sentiment classification condition, the initial sentiment score of positive reviews is 1 and that of negative reviews is -1. For the sentiment rating prediction condition, the initial sentiment scores of 5-star, 4-star, 3-star, 2-star, and 1-star reviews are 1, 0.5, 0, -0.5, and -1, respectively.

Normalizing *score*(*D*) to make

$$\begin{array}{c} \sum_{d_{i}\in D_{pos}}score\left(d_{i}\right)=1,\sum_{d_{i}\in D_{neg}}score\left(d_{i}\right)=-1 \end{array}$$ (17)

Normalizing *score*(*W*) to make

$$\begin{array}{c} \sum_{w_{j}\in W_{pos}}score\left(w_{j}\right)=1,\sum_{w_{j}\in W_{neg}}score\left(w_{j}\right)=-1 \end{array}$$ (18)

Initializing *score*(*T*) to make *score*(*t*_*h*_) = 0, 1 ≤ *h* ≤ *l*.

Normalizing *score*(*D*) in accordance with Eq (19):

$$\begin{array}{c} score\left(d_{i}\right)=\{\begin{array}{cc} \frac{score(d_{i})}{\sum_{d_{i}\in D_{pos}}score\left(d_{i}\right)} & score(d_{i})\geq 0\\ \frac{score(d_{i})}{\sum_{d_{i}\in D_{neg}}\left(-score\left(d_{i}\right)\right)} & score(d_{i})<0 \end{array} \end{array}$$ (19)

Normalizing *score*(*T*) in accordance with Eq (20):

$$\begin{array}{c} score\left(t_{h}\right)=\{\begin{array}{cc} \frac{score(t_{h})}{\sum_{t_{h}\in T_{pos}}score\left(t_{h}\right)} & score(t_{h})\geq 0\\ \frac{score(t_{h})}{\sum_{t_{h}\in T_{neg}}\left(-score\left(t_{h}\right)\right)} & score(t_{h})<0 \end{array} \end{array}$$ (20)

Normalizing *score*(*W*) in accordance with Eq (21):

$$\begin{array}{c} score\left(w_{j}\right)=\{\begin{array}{cc} \frac{score(w_{j})}{\sum_{w_{j}\in W_{pos}}score\left(w_{j}\right)} & score(w_{j})\geq 0\\ \frac{score(w_{j})}{\sum_{w_{j}\in W_{neg}}\left(-score\left(w_{j}\right)\right)} & score(w_{j})<0 \end{array} \end{array}$$ (21)

To obtain the steady sentiment score of documents, we repeat the ToD, ToT, and ToW processes until the variation amount of all *score*(*d*_*i*_) is less than 0.00001.

We renormalize *score*(*D*) using Eq (22) in order to obtain the sentiment score of documents.

$$\begin{array}{c} score\left(d_{i}\right)=\{\begin{array}{cc} \frac{score(d_{i})}{\max(score\left(d_{i}\right))} & score(d_{i})\geq 0\\ \frac{-score(d_{i})}{\min(score\left(d_{i}\right))} & score(d_{i})<0 \end{array} \end{array}$$ (22)

**Fig 4:**
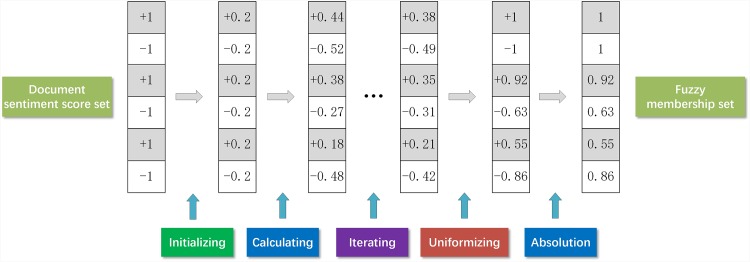
An example of initialization and normalization of sentiment score.

The absolute value |*score*(*d*_*i*_)| of the sentiment score *score*(*d*_*i*_) is defined as the fuzzy sentiment membership of a document (*d*_*i*_). An example of initialization and normalization of sentiment score can be seen in Fig 4.

### Sentiment propagation algorithm and discussions

#### Sentiment propagation algorithm

We use sentiment propagation algorithm to get the sentiment scores of documents, topics, and words in TLSPM. Specifically, for implementing sentiment propagation in the sentiment network, we construct the sentiment network and its matrix representation between documents, topics, and words. The overall sentiment propagation process in the sentiment network can be divided into toward document, toward document, and toward word. We design the sentiment propagation process by using the sentiment propagation Formulas (14–16) and the normalization of the sentiment score Formulas (17–22). Generally, we think that strong sentiment intensity of positive or negative documents make large contributions to sentiment classification, while weak sentiment intensity samples are unimportant. The sentiment intensity can reflect the fuzzy membership to the sentiment labels. Therefore we determine the absolute value of sentiment score as the fuzzy sentiment membership. Then we get the fuzzy membership set and fuzzy training document set.

The complete algorithm is described in Algorithm 1.

**Algorithm 1**: Sentiment propagation algorithm

**Input**: Training text set *D*_*train*_ = {*d*_1_, *d*_2_, ⋯, *d*_*n*_}, (1 ≤ *i* ≤ *n*), topic set *T* = {*t*_1_, *t*_2_, ⋯, *t*_*l*_}, (1 ≤ *h* ≤ *l*), word set *W* = {*w*_1_, *w*_2_, ⋯, *w*_*m*_}, (1 ≤ *j* ≤ *m*), initial sentiment score vector *score*(*W*), *score*(*T*), and *score*(*D*), weighting parameters *α*, *β*, and *γ*, *α* + *β* + *γ* = 1.

**output**: Fuzzy training document set $D_{train}^{F}$.

1 Construct the sentiment network **G** = {(*D*_*train*_, *T*, *W*), *E*};

2 Initialize sentiment score vector *score*(*D*) and *score*(*W*) using Eqs (17) and (18), initialize *score*(*T*) to make *score*(*t*_*h*_) = 0;

3 **repeat**

4 **for** 1 ≤ *i* ≤ *n* **do**

5  Calculate *score*(*d*_*i*_) using Eq (14);

6 **end**

7 Normalize *score*(*D*) using Eq (19);

8 **for** 1 ≤ *h* ≤ *l* **do**

9  Calculate *score*(*t*_*h*_) using Eq (15);

10 **end**

11 Normalize *score*(*T*) using Eq (20);

12 **for** 1 ≤ *j* ≤ *m* **do**

13  Calculate *score*(*w*_*j*_) using Eq (16);

14 **end**

15 Normalize *score*(*W*) using Eq (21);

16 **until converges**

17 Renormalize *score*(*D*) using Eq (22);

18 Calculate fuzzy membership set *S* as *s*_*i*_ = |*score*(*d*_*i*_)|;

19 Return fuzzy training document set $D_{train}^{F}$.

#### Complexity analysis and convergence

In each iteration of TLSPM, we need *O*(*n*(*n*^2^ + *l*^2^ + *m*^2^)) to update *score*(*D*) in ToD process, *O*(*l*(*n*^2^ + *l*^2^ + *m*^2^)) to update *score*(*T*) in ToT process, and *O*(*m*(*n*^2^ + *l*^2^ + *m*^2^)) to update *score*(*W*) in ToW process. The complexity in each iteration is *O*((*n* + *l* + *m*)(*n*^2^ + *l*^2^ + *m*^2^)), where *n* is the number of training documents, *l* is the number of extracted topics, and *m* is the number of words.

Now, we illustrate the convergency of the algorithm. To keep the convergence of the sentiment propagation algorithm, if any row of $\tilde{\text{P}}$, $\tilde{\text{Q}}$, $\tilde{\text{M}}$, $\tilde{\text{N}}$, $\tilde{\text{U}}$, $\tilde{\text{V}}$, $\tilde{\text{G}}$, $\tilde{\text{H}}$, and $\tilde{\text{Z}}$ is 0, we assign 1/*k* to each element in the corresponding row. In the sentiment network **G** for sentiment propagation, for any given *d*_*i*_, *t*_*h*_ or *w*_*j*_, a path constituted by other documents, topics, or words connected to it must exist. Therefore, the sum of each row in $\tilde{\text{P}}$, $\tilde{\text{Q}}$, $\tilde{\text{M}}$, $\tilde{\text{N}}$, $\tilde{\text{U}}$, $\tilde{\text{V}}$, $\tilde{\text{G}}$, $\tilde{\text{H}}$, and $\tilde{\text{Z}}$ is not 0. This indicates that the sentiment network **G** is strongly connected and the corresponding matrix **G** is irreducible. According to [39] and [40], *score*(*D*) must be able to converge to a stable value.

#### Parameter selection

In this paper, we use the validation set to determine the parameters set *θ* = {*k*, *ntopics*, *α*, *β*, *γ*}, where *k* is the selected number of sentiment neighbors, *ntopics* is the number of topics, *α* is the texts weight, *β* is the topics weight, *γ* is the words weight.

The loss function is defined as

$$\begin{array}{c} L\left(y,\hat{y}\right)=\frac{1}{2}|y-f(d,\theta )| \end{array}$$ (23)

Where *y* is the true label of *d*, $\hat{y}=f\left(d,\theta \right)$ is the prediction label by FSVM.

The parameter optimization goal is to estimate the parameters:

$$\begin{array}{c} \hat{\theta }=\underset{\theta }{\arg\min}\frac{1}{n}\sum_{i=1}^{n}L\left(y_{i},f\left(d_{i},\theta \right)\right) \end{array}$$ (24)

Where $\hat{\theta }$ is the optimal parameters set, *n* is the number of training documents.

To get the optimal parameters, we test the influence of parameters on the validation data set in the proposed approach. To test the parameters which influence the accuracy of the algorithm, we fix the other parameters remaining unchanged during the testing, test various parameters influencing the accuracy of the algorithm individually. After obtaining the optimal parameters with the best accuracy on the validation data set, we get the results on the testing data with the selecting the optimal parameters. In the experimental results and analysis section, we test the performance with varying number of neighbors, selected topics, and documents, topics, words weight, iteration times performance with varying number of neighbors on the validation data set.

## Sentiment classification

In this section, after describing the manner in which the sentiment scores and fuzzy membership of all training documents are obtained by the sentiment network and the sentiment propagation algorithm (SPA), we introduce their usage in sentiment classification by the FSVM.

In order to obtain a more accurate sentiment orientation of reviews from the testing set, we use *s*_*i*_ = |*score*(*d*_*i*_)| and obtain the fuzzy train set (*d*_1_, *y*_1_, *s*_1_), (*d*_2_, *y*_2_, *s*_2_), ⋯, (*d*_*n*_, *y*_*n*_, *s*_*n*_). Then, we use {(*d*_1_, *y*_1_, *s*_1_), (*d*_2_, *y*_2_, *s*_2_), ⋯, (*d*_*n*_, *y*_*n*_, *s*_*n*_)} to train an FSVM *f*. Large value of sentiment intensity of *d*_*i*_ indicates strong sentiment expression and high fuzzy sentiment membership degree. Therefore *d*_*i*_ makes big contribution of to sentiment classification.

The prioritization scheme of the FSVM can be formalized as

$$\begin{array}{c} \max W\left(\alpha \right)=\sum_{i=1}^{n}\alpha_{i}-\frac{1}{2}\sum_{i=1}^{n}\sum_{j=1}^{n}\alpha_{i}\alpha_{j}y_{i}y_{j}\left(d_{i}\cdot d_{j}\right) \end{array}$$ (25)

$$\begin{array}{c} subject\;to\;\sum_{i=1}^{n}\alpha_{i}y_{i}=0 \end{array}$$ (26)

$$\begin{array}{c} 0\leq \alpha_{i}\leq s_{i}C,i=1,2,\cdots ,n \end{array}$$ (27)

According to Eqs (25)–(27), we obtain the optimal solution $\alpha^{*}={\left(\alpha_{1}^{*},\alpha_{2}^{*},\cdots ,\alpha_{n}^{*}\right)}^{T}$.

In $\alpha^{*}={\left(\alpha_{1}^{*},\alpha_{2}^{*},\cdots ,\alpha_{n}^{*}\right)}^{T}$, if $\alpha_{i}^{*}>0$, the corresponding *d*_*i*_ is the support vector. If $0<\alpha_{i}^{*}<s_{i}C$, this type of support vector lies in the edge of the hyper plane. If $\alpha_{i}^{*}=s_{i}C$, this type of support vector is a misclassified sample.

An important difference between the SVM and FSVM models is that points with the same value of $\alpha_{i}^{*}$ may indicate different types of support vector because of the value of *s*_*i*_: A small value makes the sample *d*_*i*_ less important in the training and a big value makes the sample *d*_*i*_ more important to the classification [41].

In this study, we used the Gaussian kernel function $K\left(d_{i},d_{j}\right)=\exp\left(\frac{{∥d_{i}-d_{j}∥}^{2}}{2\delta^{2}}\right)$ as the kernel for constructing the FSVM classifier. The corresponding optimal solution is $\alpha^{*}={\left(\alpha_{1}^{*},\alpha_{2}^{*},\cdots ,\alpha_{n}^{*}\right)}^{T}$. Therefore, the fuzzy optimal classification is

$$\begin{array}{c} f^{*}\left(x\right)=sgn\left\{\sum_{j=1}^{n}\alpha_{j}^{*}y_{j}K\left(d_{i},d_{j}\right)+b^{*}\right\} \end{array}$$ (28)

$$\begin{array}{c} b^{*}=y_{i}-\sum_{j=1}^{n}\alpha_{j}^{*}y_{j}K\left(d_{i},d_{j}\right),j\in \left\{j|0<\alpha_{j}^{*}<s_{j}C\right\} \end{array}$$ (29)

For the two-class sentiment classification task, we use the positive tendency reviews as the positive category samples and negative tendency reviews as the negative category samples to train the FSVM. For the sentiment rating prediction task, the only difference is that we use one of the classes as the positive category and the remaining classes as the negative category to train the FSVM (one versus the rest). For example, in the K-class classification task, we use one of the classes as the positive and the remaining K-1 classes as the negative category to train the FSVM. Finally, we obtain K classifiers {*f*_1_, *f*_2_, ⋯, *f*_*K*_}. Therefore, each test sample *d*_*i*_ has K results:

$$\begin{array}{c} \left\{\left(f_{1}\left(d_{i}\right),\varepsilon \left(f_{1}\left(d_{i}\right)\right)\right),\cdots ,\left(f_{K}\left(d_{i}\right),\varepsilon \left(f_{K}\left(d_{i}\right)\right)\right)\right\} \end{array}$$ (30)

where *f*_*K*_(*d*_*i*_) is the result of the *Kth* classifier and *ε*(*f*_*K*_(*d*_*i*_)) is the confidence of the *Kth* classifier. Finally, we select the corresponding label of *max*{*ε*(*f*_1_(*d*_*i*_)), *ε*(*f*_2_(*d*_*i*_)), ⋯, *ε*(*f*_*K*_(*d*_*i*_))} as the final label of *d*_*i*_.

## Experimental design

### Data sets

We constructed experiments using eight two-class sentiment classification data sets and seven sentiment rating prediction data sets. Books (2), DVD (2), Electronics (2), and Kitchen (2) are review sets from Blitzer et al. [42]. Each data set contains 2000 reviews, of which 1000 are positive and 1000 are negative. Notebook (2), Hotel (2) and E-commerce (2) are Chinese review sets from Tan et al. [43]. Each data set has 4000 reviews, of which 2000 are positive and 2000 are negative. Movie (2) is a review set from Pang et al. [27]. This data set contains 50000 reviews, of which 25000 are positive and 25000 are negative. The details of the eight two-class sentiment classification data sets are shown in Table 1. All eight two-class sentiment classification data sets are balanced. Books (4), DVD (4), Electronics (4), Kitchen (4) are review sets from Blitzer et al. [42]. Hotel (5) and MP3 (5) are review sets from Wang et al. [44]. Movie (5) is a review set from Pang et al. [27]. Table 2 shows the sentiment rating distributions of seven sentiment rating prediction data sets. In Table 2, it can be seen that all the sentiment rating prediction data sets, except MP3 (5), are primary balanced. The sentiment ratings distribution of the MP3 (5) data set is unbalanced: the 5-star ranking has the most reviews, and 2-star and 3-star rankings have the least reviews.

**Table 1:** Positive and negative reviews in eight two-class sentiment classification data sets.

**Category**	**Books(2)**	**DVD(2)**	**Electronics(2)**	**Kitchen(2)**
Positive	1000	1000	1000	1000
Negative	1000	1000	1000	1000
Total	2000	2000	2000	2000
**Category**	**Notebook(2)**	**Hotel(2)**	**E-commerce(2)**	**Movie(2)**
Positive	2000	2000	2000	25000
Negative	2000	2000	2000	25000
Total	4000	4000	4000	50000

**Table 2:** Ratings distributions of seven sentiment rating prediction data sets.

Data set	Reviews	1-star	2-star	3-star	4-star	5-star
Books (4)	5501	1386	1400	0	1381	1344
DVD (4)	5118	1321	1244	0	1273	1280
Electronics (4)	5901	1468	1466	0	1483	1484
Kitchen (4)	5149	1300	1313	0	1262	1274
Movie (5)	15414	2899	3012	3100	3047	3356
Hotel (5)	21567	3985	4180	4566	4215	4621
MP3 (5)	31000	4404	2077	2531	7508	14479

### Text representation and processing

For 12 English data sets, we use the fisher feature selection method [45] to choose the top-800 effective features after removing the stop words. The top 15 features from the Books (2) data set are “poor, fan, lack, repeat, evidence, negative, disappointment, completely, democracy, classic, level, rich, strange, great, and intelligence”. If a word appears in the text, the weight is 1; otherwise the weight is 0. Each review is represented as a bag of words, and then, expressed as a vector space model.

For the three Chinese data sets (Notebook (2), Hotel (2), and E-commerce (2)), we first use an MMSEG segmentation algorithm (http://technology.chtsai.org/mmseg) for segmentation. MMSEG is a word identification system for mandarin Chinese text based on two variants of the maximum matching algorithm. Then, we remove stop words taken from the Chinese stopping words vocabulary. Top-1000 features are selected by fisher feature selection method [45] for each Chinese data set. For example, the top 15 features from the Notebook (2) data set are as follows: 外观 (appearance), 不错 (not bad), 配置 (configuration), 漂亮 (beautiful), 很好 (good), 性能 (performance), 麻烦 (trouble), 系統 (system), 满意 (satisfy), 便宜 (cheap), 舒服 (comfortable), 价位 (price), 轻便 (light), 做工 (workmanship), 喜欢 (love)”. Then we express each text as a vector space model, and the feature weight is determined by Boolean value.

We use the JGibbLDA model (http://jgibblda.sourceforge.net) to extract topics in the document. -Alpha is set as 50/*ntopics*, -twords is set as 50, -savestep is set as 200, -niters is set as 1000, -beta is set as 0.1.

### Evaluation metrics

In this study, the evaluation metrics [46] for two-class sentiment classification are shown in Table 3.

$$\begin{array}{c} RN\left(\text{Recall}\right)=\frac{d}{b+d}\times 100\% \end{array}$$ (31)

$$\begin{array}{c} PN\left(\text{Precision}\right)=\frac{d}{c+d}\times 100\% \end{array}$$ (32)

$$\begin{array}{c} FN\left(\text{F1-measure}\right)=\frac{2\times RN\times PN}{RN+PN}\times 100\% \end{array}$$ (33)

$$\begin{array}{c} RP\left(\text{Recall}\right)=\frac{a}{a+c}\times 100\% \end{array}$$ (34)

$$\begin{array}{c} PP\left(\text{Precision}\right)=\frac{a}{a+b}\times 100\% \end{array}$$ (35)

$$\begin{array}{c} FP\left(\text{F1-measure}\right)=\frac{2\times RP\times PP}{RP+PP}\times 100\% \end{array}$$ (36)

$$\begin{array}{c} Acc\left(\text{Accuracy}\right)=\frac{a+d}{a+b+c+d}\times 100\% \end{array}$$ (37)

**Table 3:** Confusion matrix of two-class sentiment classification results.

	actual positive	actual negative
positive prediction	a	b
negative prediction	c	d

For sentiment rating prediction, we used accuracy and mean square error (MSE) as the evaluation metrics. The calculation method is

$$\begin{array}{c} Acc=\frac{n(right\;answer)}{N-n} \end{array}$$ (38)

$$\begin{array}{c} MSE=\frac{\sum_{i=n+1}^{N}{\left(answer_{i}-result_{i}\right)}^{2}}{N-n} \end{array}$$ (39)

where *n*(*right* *answer*) is the number of samples, the output ratings of which are in accordance with the original ratings, *N* − *n* is the total number of test samples, *i* is the number of test samples, *answer*_*i*_ is the sentiment rating of the original rating of *d*_*i*_, and *result*_*i*_ is the output rating.

Following most experiment results that were used in studies in the literature, we extract 60% as the training set, 20% as the validation set, and the remaining 20% as the testing set by using stratified sampling [22, 47]. We train the FSVM with TLSPM on the training set, tune parameters on the validation set, and evaluate the effectiveness on the testing set.

### Baselines

To verify the validity of TLSPM, we design the following comparison test. The fuzzy membership that generated by Lexicon, Centroid, S-type, Compact, and TLSPM are the input of FSVM. The classification results of FSVM can verify the effectiveness of the listed five fuzzy membership determining methods.

- **SVM**: Use LIBSVM (http://www.csie.ntu.edu.tw/∼cjlin/libsvm) with a linear kernel and default parameters [48].
- **Lexicon**:. SentiWordNet (http://sentiwordnet.isti.cnr.it) 3.0 is a lexical resource publicly available for research purposes and is an improved version of SentiWordNet 1.0 [49]. We used the positivity, negativity, and neutrality scores that are annotated by SentiWordNet3.0. We used the positive sentiment scores minus the negative sentiment scores of all the terms in the review as the final score of a review. Finally, the fuzzy membership is determined by the absolute value of sentiment score.
- **Centroid**: Fuzzy membership determining mechanism based on distance to the class centroid [8]. The definition of the centroid of the training set *S* is $\hat{d}=\frac{1}{n}\sum_{i=1}^{n}d_{i}$ and the distance between sample *d*_*i*_ and class centroid $\hat{d}$ is defined as
  $$\begin{array}{c} dis\left(d_{i},\hat{d}\right)=\frac{d_{i}\cdot \hat{d}}{\left|d_{i}|\times |\hat{d}\right|} \end{array}$$ (40)
  The fuzzy membership *s*_*i*_ of *d*_*i*_ is
  $$\begin{array}{c} s_{i}=\mu \left(d_{i}\right)=1-\frac{dis(d_{i},\hat{d})}{r} \end{array}$$ (41)
  where *r* is the radius of the class and $r=\max\{dis\left(d_{i},\hat{d}\right)\}.$
- **S-type**: Lin and Wang [41] first calculated the distance between *d*_*i*_ and class centroid $\hat{d}$, $dis\left(d_{i},\hat{d}\right)=\sqrt{\sum_{k=1}^{m}{\left(d_{ik}-{\hat{d}}_{k}\right)}^{2}}$, where *m* was the dimension of *d*_*i*_, and set the parameters *b*_1_ = 0.1, *b*_2_ = 0.5, and *b*_3_ = 0.9. They rehabilitated Zadeh’s proposed standard for S-function transformation and obtained the fuzzy membership function
  $$\begin{array}{c} s_{i}=\mu \left(dis\left(d_{i},\hat{d}\right);b_{1},b_{2},b_{3}\right)=\\ \left\{ \begin{array}{c} 1\\ 1-2{\left[\left(dis\left(d_{i},\hat{d}\right)-b_{1}\right)/\left(b_{3}-b_{1}\right)\right]}^{2}\\ 2{\left[\left(dis\left(d_{i},\hat{d}\right)-b_{1}\right)/\left(b_{3}-b_{1}\right)\right]}^{2}\\ 0 \end{array}\begin{array}{c} dis\left(d_{i},\hat{d}\right)<b_{1}\\ b_{1}\leq dis\left(d_{i},\hat{d}\right)<b_{2}\\ b_{2}\leq dis\left(d_{i},\hat{d}\right)<b_{3}\\ dis\left(d_{i},\hat{d}\right)\geq b_{3} \end{array}\right. \end{array}$$ (42)
  where $dis(d_{i},\hat{d})$ is the distance between *d*_*i*_ and class centroid $\hat{d}$, *b*_1_, *b*_2_ and *b*_3_ are predefined parameters, and *b*_2_ = (*b*_1_ + *b*_3_)/2. If $dis\left(d_{i},\hat{d}\right)=b_{2}$, *s*_*i*_ = 0.5.
- **Compact**: Batuwita et al. [13] defined the fuzzy membership as
  $$\begin{array}{c} s_{i}=f\left(\mu \left(d_{i}\right),\mu_{k}\left(d_{i},\hat{d}\right)\right) \end{array}$$ (43)
  where *μ*(*d*_*i*_) is the membership of *d*_*i*_ and belongs to $\hat{d}$ and $\mu_{k}\left(d_{i},\hat{d}\right)$ is a fuzzy connectivity membership to the centroid and is determined by
  $$\begin{array}{c} \mu_{k}\left(d_{i},\hat{d}\right)=\max_{\rho \left(d_{i},\hat{d}\right)\in P\left(d_{i},\hat{d}\right)}\left[\min\left(\mu_{k}\left(e_{1},e_{2}\right),\cdots ,\mu_{k}\left(e_{m-1},e_{m}\right)\right)\right] \end{array}$$ (44)
  where $\rho (d_{i},\hat{d})$ is a path from *d*_*i*_ to $\hat{d}$, on which each point is represented by *e*_1_, *e*_2_, ⋯, *e*_*m*_, where *e*_1_ = *d*_*i*_ and $e_{m}=\hat{d}$. $P(d_{i},\hat{d})$ is the set of all the paths from *d*_*i*_ to $\hat{d}$.
  We define *s*_*i*_ using Eq (45):
  $$\begin{array}{c} s_{i}=f\left(\mu \left(d_{i}\right),\mu_{k}\left(d_{i},\hat{d}\right)\right)=\mu \left(d_{i}\right)\times \mu_{k}\left(d_{i},\hat{d}\right) \end{array}$$ (45)
  *μ*(*d*_*i*_) is calculated by Eq (41).
- **TLSPM**: Our proposed three-layer sentiment propagation model.

## Experimental results and analysis

### Comparing results and analysis

In order to validate the effectiveness of the presented TLSPM, we designed experiments using 15 real-world sentiment data sets. At the same time, we compared TLSPM with SVM and four other fuzzy membership determination methods.

**Fig 5:**
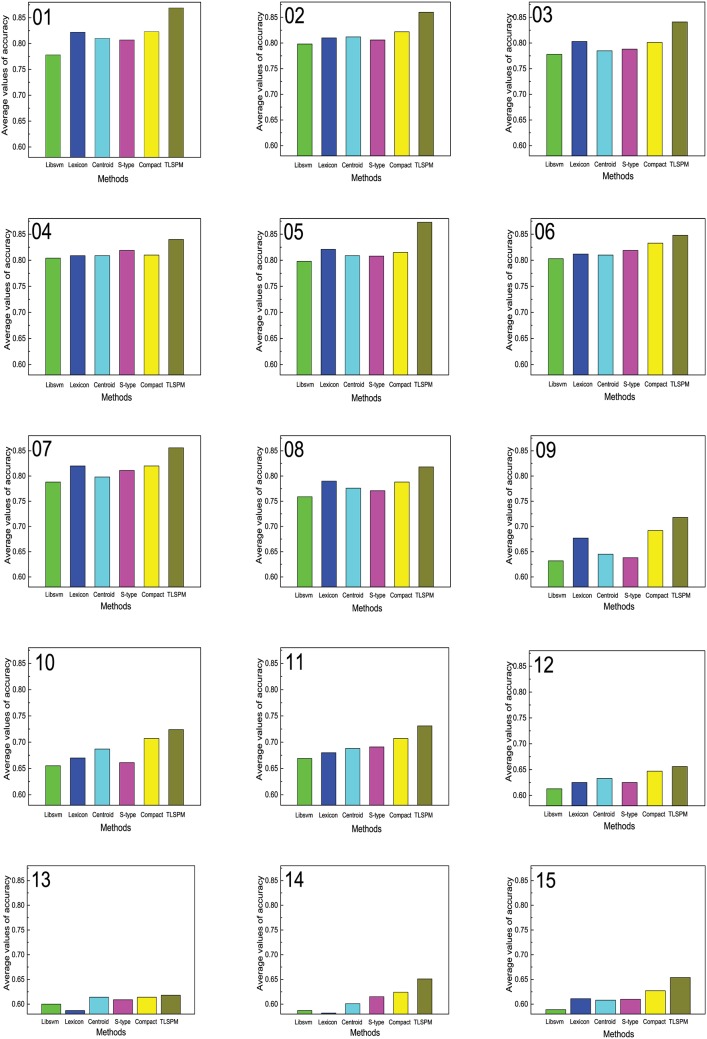
Accuracy comparison of different methods on the testing set. (01) Books (2); (02) DVD (2); (03) Electric (2); (04) Kitchen (2); (05) Notebook (2); (06) Hotel (2); (07) E-commerce (2); (08) Movie (2); (09) Books (4); (10) DVD (4); (11) Electric (4); (12) Kitchen (4); (13) Movie (5); (14) Hotel (5); (15) MP3 (5).

The comparative experimental results on the testing data can be seen in Tables 4–12. Fig 5 shows the accuracy comparison of the different methods on the testing data. The parameters *k*, *ntopics*, *α*, *β*, and *γ* having the best accuracy on the validation set are shown in Table 13.

**Table 4:** Experimental results for Books (2) data set.

Methods	RN	PN	FN	RP	PP	FP	Acc
SVM	0.782	0.776	0.779	0.774	0.780	0.777	0.778
Lexicon	0.824	0.820	0.822	0.819	0.823	0.821	0.822
Centroid	0.819	0.805	0.812	0.801	0.816	0.808	0.810
S-type	0.815	0.801	0.808	0.798	0.812	0.805	0.807
Compact	0.821	0.824	0.823	0.825	0.822	0.823	0.823
TLSPM	0.867	0.870	0.868	0.870	0.867	0.869	0.869

**Table 5:** Experimental results for DVD (2) data set.

Methods	RN	PN	FN	RP	PP	FP	Acc
SVM	0.785	0.806	0.795	0.811	0.790	0.801	0.798
Lexicon	0.815	0.806	0.811	0.804	0.813	0.808	0.810
Centroid	0.802	0.818	0.810	0.821	0.806	0.813	0.812
S-type	0.817	0.799	0.808	0.794	0.800	0.797	0.806
Compact	0.824	0.820	0.822	0.819	0.823	0.821	0.822
TLSPM	0.868	0.854	0.861	0.851	0.867	0.858	0.860

**Table 6:** Experimental results for Electronic (2) data set.

Methods	RN	PN	FN	RP	PP	FP	Acc
SVM	0.787	0.772	0.780	0.768	0.783	0.775	0.778
Lexicon	0.806	0.801	0.804	0.800	0.805	0.802	0.803
Centroid	0.795	0.779	0.787	0.774	0.791	0.782	0.785
S-type	0.776	0.795	0.785	0.800	0.782	0.791	0.788
Compact	0.789	0.808	0.798	0.812	0.794	0.803	0.801
TLSPM	0.837	0.844	0.840	0.845	0.838	0.842	0.841

**Table 7:** Experimental results for Kitchen (2) data set.

Methods	RN	PN	FN	RP	PP	FP	Acc
SVM	0.800	0.807	0.803	0.808	0.802	0.805	0.804
Lexicon	0.811	0.808	0.809	0.807	0.810	0.809	0.809
Centroid	0.806	0.810	0.808	0.811	0.807	0.809	0.809
S-type	0.815	0.822	0.818	0.823	0.817	0.820	0.819
Compact	0.804	0.813	0.808	0.815	0.808	0.811	0.810
TLSPM	0.836	0.843	0.839	0.844	0.837	0.841	0.840

**Table 8:** Experimental results for Notebook (2) data set.

Methods	RN	PN	FN	RP	PP	FP	Acc
SVM	0.807	0.793	0.800	0.789	0.804	0.796	0.798
Lexicon	0.822	0.820	0.821	0.820	0.822	0.821	0.821
Centroid	0.813	0.807	0.810	0.805	0.812	0.808	0.809
S-type	0.806	0.808	0.807	0.809	0.807	0.808	0.808
Compact	0.815	0.814	0.815	0.814	0.815	0.814	0.815
TLSPM	0.868	0.876	0.872	0.877	0.869	0.873	0.873

**Table 9:** Experimental results for Hotel (2) data set.

Methods	RN	PN	FN	RP	PP	FP	Acc
SVM	0.805	0.801	0.803	0.800	0.804	0.802	0.803
Lexicon	0.808	0.814	0.811	0.816	0.809	0.813	0.812
Centroid	0.808	0.810	0.809	0.811	0.809	0.810	0.810
S-type	0.818	0.819	0.818	0.819	0.818	0.819	0.819
Compact	0.827	0.836	0.832	0.838	0.829	0.833	0.833
TLSPM	0.847	0.849	0.848	0.849	0.847	0.848	0.848

**Table 10:** Experimental results for E-commerce (2) data set.

Methods	RN	PN	FN	RP	PP	FP	Acc
SVM	0.785	0.789	0.787	0.790	0.786	0.788	0.788
Lexicon	0.815	0.823	0.819	0.825	0.817	0.821	0.820
Centroid	0.795	0.799	0.797	0.800	0.796	0.798	0.798
S-type	0.813	0.809	0.811	0.808	0.812	0.810	0.811
Compact	0.822	0.818	0.820	0.817	0.822	0.820	0.820
TLSPM	0.844	0.864	0.854	0.867	0.848	0.857	0.856

**Table 11:** Experimental results for Movie (2) data set.

Methods	RN	PN	FN	RP	PP	FP	Acc
SVM	0.739	0.769	0.754	0.778	0.749	0.763	0.759
Lexicon	0.784	0.793	0.788	0.796	0.786	0.791	0.790
Centroid	0.773	0.777	0.775	0.778	0.774	0.776	0.776
S-type	0.767	0.772	0.770	0.774	0.769	0.771	0.771
Compact	0.791	0.787	0.789	0.786	0.790	0.788	0.788
TLSPM	0.830	0.810	0.820	0.805	0.826	0.815	0.818

**Table 12:** Experimental results for seven sentiment rating prediction data sets.

Data set	Metrics	SVM	Lexicon	Centroid	S-type	Compact	TLSPM
Books (4)	Accuracy	0.632	0.677	0.645	0.638	0.692	0.718
	MSE	1.650	1.421	1.578	1.589	1.133	0.986
DVD (4)	Accuracy	0.655	0.670	0.687	0.661	0.707	0.724
	MSE	1.510	1.415	1.298	1.346	1.058	0.891
Electronic (4)	Accuracy	0.669	0.680	0.688	0.691	0.707	0.731
	MSE	0.128	0.115	0.106	0.964	0.887	0.778
Kitchen(4)	Accuracy	0.613	0.625	0.633	0.625	0.647	0.656
	MSE	1.433	1.320	1.118	1.192	1.103	0.865
Movie (5)	Accuracy	0.600	0.587	0.614	0.609	0.614	0.618
	MSE	1.511	1.602	1.358	1.440	1.318	1.115
Hotel (5)	Accuracy	0.570	0.582	0.601	0.615	0.624	0.651
	MSE	1.710	1.650	1.622	1.540	1.308	1.103
MP3 (5)	Accuracy	0.589	0.611	0.608	0.610	0.627	0.654
	MSE	1.654	1.558	1.549	1.365	1.338	1.204

**Table 13:** Best accuracy with *k*, *ntopics*, *α*, *β* and *γ* on the validation set by TLSPM.

Data sets	Acc	*k*	*ntopics*	*α*	*β*	*γ*
Books(2)	0.867	35	50	0.4	0.3	0.3
DVD(2)	0.864	35	50	0.3	0.5	0.2
Electronic(2)	0.838	35	60	0.4	0.3	0.3
Kitchen(2)	0.842	35	50	0.4	0.3	0.3
Notebook (2)	0.870	35	50	0.3	0.3	0.4
Hotel(2)	0.845	30	50	0.4	0.3	0.3
E-commerce (2)	0.854	35	50	0.5	0.3	0.2
Movie (2)	0.820	25	50	0.4	0.3	0.3
Books(4)	0.715	35	50	0.3	0.3	0.4
DVD(4)	0.720	35	60	0.4	0.3	0.3
Electronic(4)	0.730	35	50	0.6	0.2	0.2
Kitchen(4)	0.651	35	70	0.4	0.3	0.3
Movie (5)	0.620	35	50	0.4	0.4	0.2
Hotel (5)	0.654	35	50	0.3	0.2	0.5
MP3(5)	0.650	30	50	0.4	0.3	0.3

In Tables 4–12 and Fig 5, we can see that:

1. The accuracies of the six methods (SVM, Lexicon, Centroid, S-type, Compact, and TLSPM) on the seven sentiment rating prediction data sets are lower than on the eight two-class data sets.
2. As compared with using the SVM method directly, the accuracy of the five fuzzy membership determination methods improves greatly; for example, the Lexicon, Centroid, S-type, Compact, and TLSPM methods improved 0.044, 0.032, 0.029, 0.045, and 0.091, respectively on the Books (2) data set.
3. The accuracy of the Compact method is higher than that of the Centroid and S-type methods on 14 sentiment data sets, but not on the Kitchen (2) data set. For example, the Compact method improved 0.016 and 0.013 over the Centroid and S-type methods on the Electronic (2) data set.
4. TLSPM behaves better than Lexicon, Centroid, S-type, and Compact methods on 15 data sets, for example, TLSPM improved 0.036, 0.038, 0.029, and 0.015 on the Hotel (2) data set.

These results can be concluded as below.

1. As compared with two-class sentiment classification, sentiment rating prediction is a more challenging task, because it must not only judge the sentiment orientations of reviews, but also measure their intensity.
2. SVM has been successfully applied to sentiment classification, but it is sensitive to irrelevant and noisy training samples. The FSVM sentiment classification results that using sentiment score as fuzzy membership are very stable and robust.
3. Although TLSPM is more complex than the other methods, it can achieve more accurate sentiment score of documents. Clearly, the fuzzy membership of documents should be determined by using the semantic relations between the documents, topics, and words other than three universal spatial location fuzzy membership determining methods.

### Performance with varying number of neighbors

**Fig 6:**
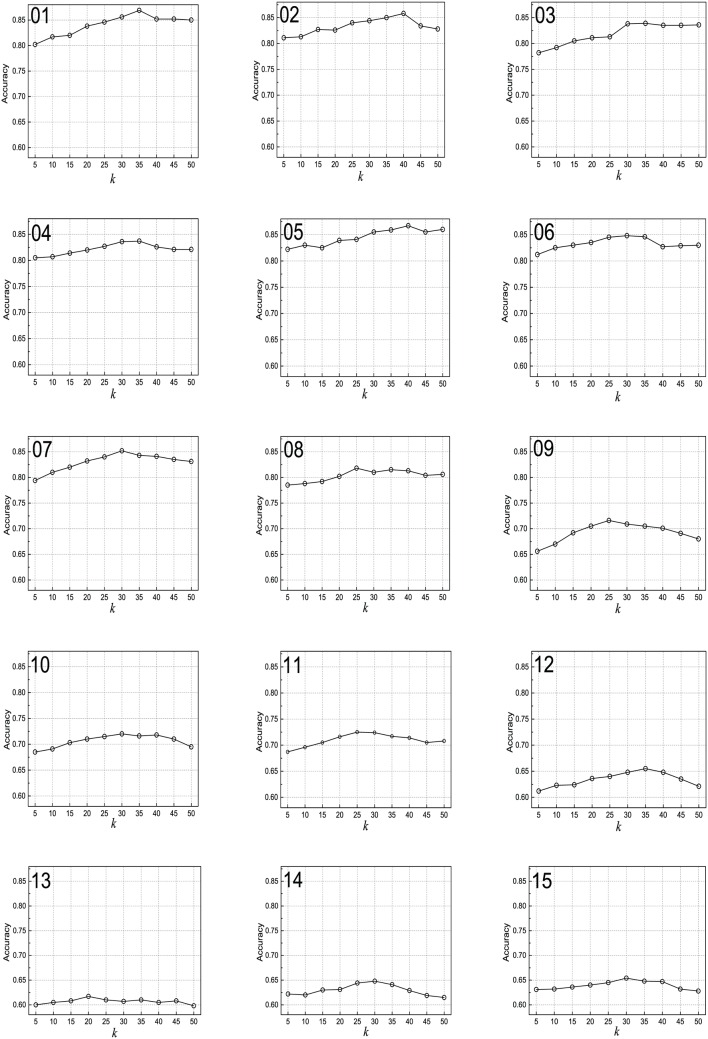
Performance with varying values of *k*. (01) Books (2); (02) DVD (2); (03) Electric (2); (04) Kitchen (2); (05) Notebook (2); (06) Hotel (2); (07) E-commerce (2); (08) Movie (2); (09) Books (4); (10) DVD (4); (11) Electric (4); (12) Kitchen (4); (13) Movie (5); (14) Hotel (5); (15) MP3 (5).

The second focus of the research was to study the performance with varying number of neighbors on the validation set. *ntopics* is set as 50, *α* is set as 0.4, *β* is set as 0.3, and *γ* is set as 0.3, we test the accuracy variation when the value of *k* increases from 5 to 50. The experimental results are given in Fig 6. It can be clearly seen that when the value of *k* changes from 5 to 50, the accuracy first increases and then stabilizes on (03) Electric (2), (08) Movie (2) and (11) Electric (4) data sets. The accuracy for the remaining data sets first increases and then decreases. Meanwhile, the accuracy of the seven sentiment rating prediction data sets is lower than that of the eight two-class sentiment data sets. The sentiment score of document, topic, or word is likely to be influenced of noise if *k* is too small. Instead, the sentiment score will be influenced of irrelevant neighbors if the selected number of neighbors is too big.

### Iteration times performance with varying number of neighbors

**Fig 7:**
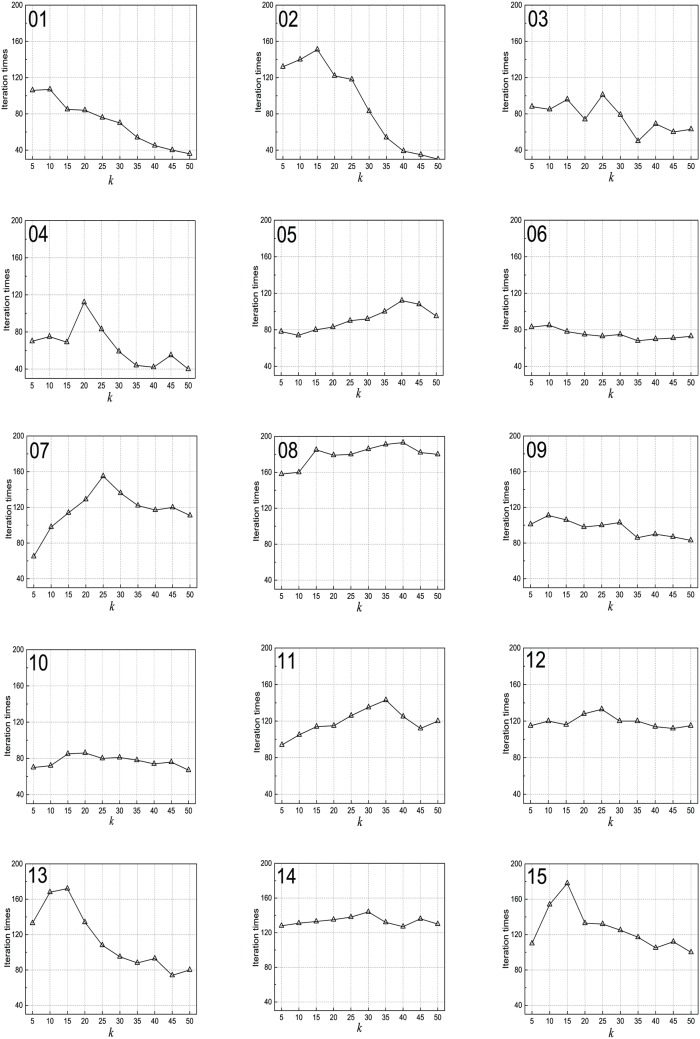
Maximum iterations for different values of *k*. (01) Books (2); (02) DVD (2); (03) Electric (2); (04) Kitchen (2); (05) Notebook (2); (06) Hotel (2); (07) E-commerce (2); (08) Movie (2); (09) Books (4); (10) DVD (4); (11) Electric (4); (12) Kitchen (4); (13) Movie (5); (14) Hotel (5); (15) MP3 (5).

To test the iteration times performance with varying neighbors when the value of *k* changes from 5 to 50, *α* is set as 0.4, *β* is set as 0.3, *γ* is set as 0.3, and *ntopics* is set as 50. The results can be seen in Fig 7. The curves of (02) DVD (2), (04) Kitchen (2), (07) E-commerce (2), (13) Movie (5), and (15) MP3 (5) are very similar: the iteration times first increase and then decrease. The only difference is that the *k* values that obtain the maximum value are different. The curves of the remaining data sets are very similar, and are not very sensitive to the number of selected neighbors *k*. This indicates that TLSPM can converge quickly and a large value of *k* frequently leads to fast convergence.

### Performance with varying selected topics

**Fig 8:**
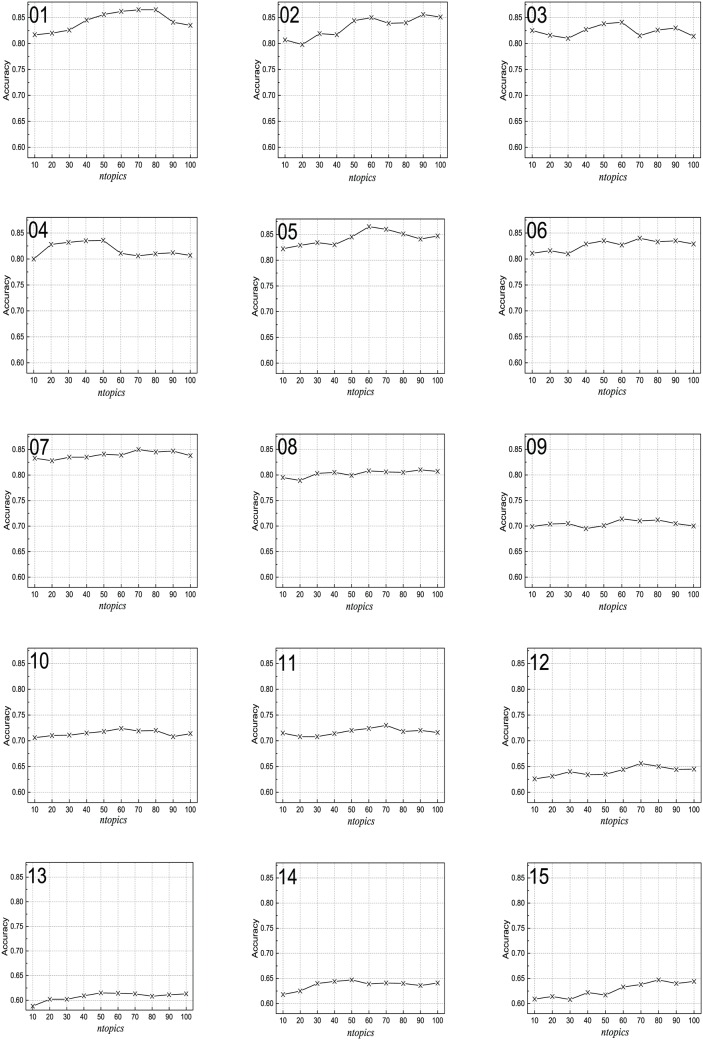
Performance with varying values of *ntopics*. (01) Books (2); (02) DVD (2); (03) Electric (2); (04) Kitchen (2); (05) Notebook (2); (06) Hotel (2); (07) E-commerce (2); (08) Movie (2); (09) Books (4); (10) DVD (4); (11) Electric (4); (12) Kitchen (4); (13) Movie (5); (14) Hotel (5); (15) MP3 (5).

In order to test the accuracy variation when the value of *ntopics* changes from 10 to 100, *k* is set as 35, *α* is set as 0.4, *β* is set as 0.3, and *γ* is set as 0.3. The results for the 15 sentiment data sets can be seen in Fig 8. As shown in Fig 8, the curves of the accuracy of the 15 data sets are very similar. The only difference is that the accuracy of the seven sentiment rating prediction sets is lower than that of the eight two-class sentiment data sets. The accuracy first increases and then decreases with the increase number of selected topics.

### Performance with varying documents, topics, words weight

**Fig 9:**
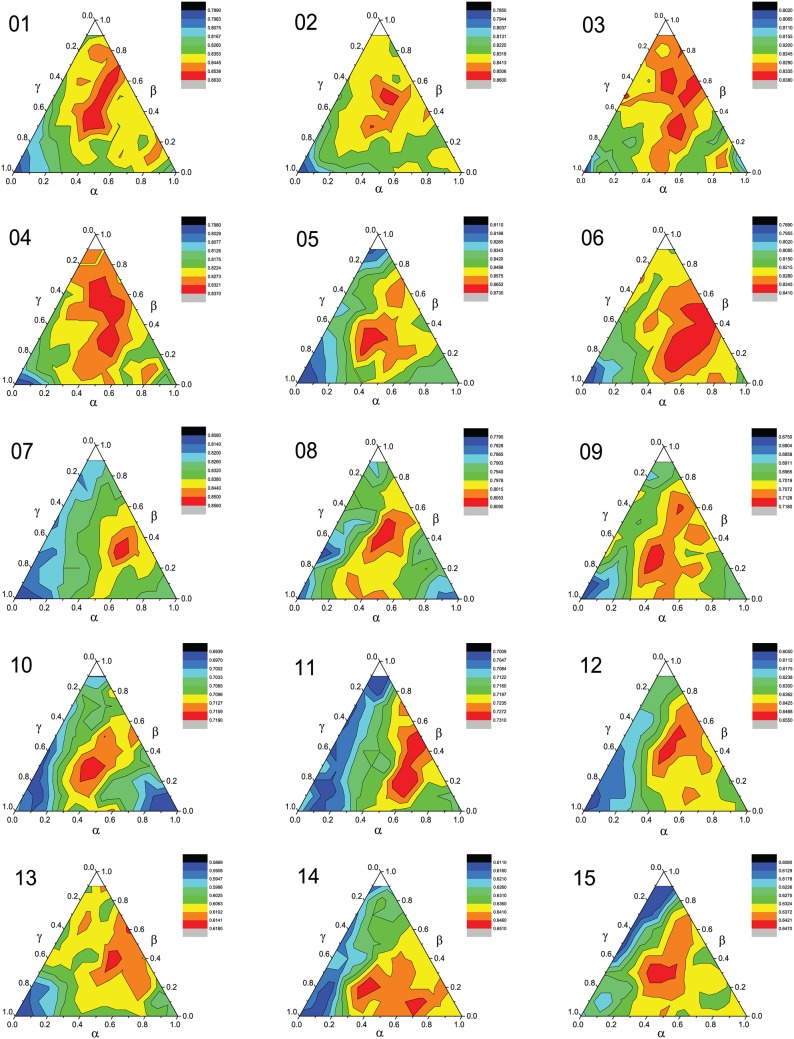
Accuracy ternary contour distribution for different values of the documents, topics, words weight. (01) Books (2); (02) DVD (2); (03) Electric (2); (04) Kitchen (2); (05) Notebook (2); (06) Hotel (2); (07) E-commerce (2); (08) Movie (2); (09) Books (4); (10) DVD (4); (11) Electric (4); (12) Kitchen (4); (13) Movie (5); (14) Hotel (5); (15) MP3 (5).

To further demonstrate the performance with varying documents, topics, words weight, *k* is set as 35, *ntopics* is set as 50, restricted conditions are *γ* = 1 − *α* − *β* and *α* + *β* < 1. As shown in Fig 9, we can see that different data sets have different accuracy ternary contour distributions, and the different weights value affect the final sentiment classification results. Specifically, when *α*, *β*, and *γ* have the same value in principle, we get the maximum accuracy. This indicates that words, topics, and documents are all the seem important in determining fuzzy membership of document.

## Conclusions and future work

In this paper, a new framework of determining the fuzzy sentiment membership of documents is adopted for sentiment classification task. Our main findings and contributions include the following items. The sentiment score can describe the sentiment orientation and sentiment intensity in great detail. Therefore, sentiment score determination methods are very useful for fine-grained sentiment analysis. Our experiments verified this finding and a better sentiment classification result is achieved by using the sentiment score as fuzzy sentiment membership. SVM has been successfully applied to sentiment classification, but it is sensitive to irrelevant and noisy training samples. Our experiments show that the FSVM model can resolve this problem by assigning different fuzzy memberships to different samples, and different fuzzy membership determination methods lead to different classification results. As is known, sentiment expression is very domain-specific. The same word may have different sentiment orientations and intensity in different domains. Therefore, it is not appropriate to determine the sentiment score of reviews by using an universal sentiment lexicon. The proposed three-layer sentiment propagation model determines the sentiment score of reviews by using the semantic relationships of documents, topics, and words. Therefore, it performs better than three universal spatial location fuzzy membership determining methods (Centroid, S-type, and Compact).

For large-scale sentiment classification task, TLSPM need to solve the following problems: hyper-high dimensional space, massive operation time, and so on. To reduce storage space and running time, we plan to use the partition and combination of the adjacent matrices between documents, topics, and words from MapReduce framework. In the later research, we will promote TLSPM to decrease the algorithm complexity in the matrix and validate our method in big data sets.

## References

[1] Tang D, Qin B, Liu T. Document modeling with gated recurrent neural network for sentiment classification. In: Proceedings of the 2015 Conference on Empirical Methods in Natural Language Processing; 2015. p. 1422–1432.

[2] Liu Y, Yu X, Chen Z, Liu B. Sentiment analysis of sentences with modalities. In: Proceedings of the 2013 International Workshop on Mining Unstructured Big Data Using Natural Language Processing. ACM; 2013. p. 39–44.

[3] Ranco G, Aleksovski D, Caldarelli G, Grcar M, Mozetic I. The effects of Twitter sentiment on stock price returns. PloS one. 2015;10(9):e0138441 10.1371/journal.pone.0138441 26390434 PMC4577113

[4] Agarwal B, Mittal N, Bansal P, Garg S. Sentiment analysis using common-sense and context information. Computational intelligence and neuroscience. 2015;2015:30 10.1155/2015/715730 PMC4381572 25866505

[5] Zhao C, Wang S, Li D. Fuzzy sentiment membership determining for sentiment classification. In: Proceedings of the 2014 IEEE International Conference on Data Mining Workshop. IEEE Computer Society; 2014. p. 1191–1198.

[6] Liu D, Li T, Liang D. Incorporating logistic regression to decision-theoretic rough sets for classifications. International Journal of Approximate Reasoning. 2014;55(1):197–210. 10.1016/j.ijar.2013.02.013

[7] Singh N, Mishra RK. Unintentional activation of translation equivalents in bilinguals leads to attention capture in a cross-modal visual task. PloS one. 2015;10(3):e0120131 10.1371/journal.pone.0120131 25775184 PMC4361716

[8] Lin CF, Wang SD. Fuzzy support vector machines. IEEE Transactions on Neural Networks. 2002;13(2):464–471. 10.1109/72.991432 18244447

[9] Yu H, Liu Z, Wang G. An automatic method to determine the number of clusters using decision-theoretic rough set. International Journal of Approximate Reasoning. 2014;55(1):101–115. 10.1016/j.ijar.2013.03.018

[10] Liu D, Li T, Li H. A multiple-category classification approach with decision-theoretic rough sets. Fundamenta Informaticae. 2012;115(2-3):173–188. 10.1007/978-3-642-16248-0_95

[11] Zou Q, Zeng J, Cao L, Ji R. A novel features ranking metric with application to scalable visual and bioinformatics data classification. Neurocomputing. 2016;173:346–354. 10.1016/j.neucom.2014.12.123

[12] Neagu DC, Guo G, Trundle PR, Cronin M. A comparative study of machine learning algorithms applied to predictive toxicology data mining. Alternatives to laboratory animals: ATLA. 2007;35(1):25–32. 17411348 10.1177/026119290703500119

[13] Batuwita R, Palade V. FSVM-CIL: Fuzzy support vector machines for class imbalance learning. IEEE Transactions on Fuzzy Systems. 2010;18(3):558–571. 10.1109/TFUZZ.2010.2042721

[14] Liang J, Zhou X, Guo L, Bai S. Feature selection for sentiment classification using matrix factorization. In: Proceedings of the 24th International Conference on World Wide Web. ACM; 2015. p. 63–64.

[15] Vo DT, Zhang Y. Target-dependent twitter sentiment classification with rich automatic features. In: Proceedings of the Twenty-Fourth International Joint Conference on Artificial Intelligence (IJCAI 2015); 2015. p. 1347–1353.

[16] Yu Z, Wong RK, Chi CH, Chen F. A semi-supervised learning approach for microblog sentiment slassification. In: 2015 IEEE International Conference on Smart City/SocialCom/SustainCom (SmartCity). IEEE; 2015. p. 339–344.

[17] Turney PD. Thumbs up or thumbs down?: Semantic orientation applied to unsupervised classification of reviews. In: Proceedings of the 40th Annual Meeting on Association for Computational Linguistics. Association for Computational Linguistics; 2002. p. 417–424.

[18] Ohana B, Tierney B. Sentiment classification of reviews using SentiWordNet. In: Proceedings of the 9th. Annual Information Technology Telecommunications Conference; 2009. p. 13–21.

[19] Kanayama H, Nasukawa T. Fully automatic lexicon expansion for domain-oriented sentiment analysis. In: Proceedings of the 2006 Conference on Empirical Methods in Natural Language Processing. Association for Computational Linguistics; 2006. p. 355–363.

[20] Wan X. Co-training for cross-lingual sentiment classification. In: Proceedings of the Joint Conference of the 47th Annual Meeting of the ACL and the 4th International Joint Conference on Natural Language Processing of the AFNLP. Association for Computational Linguistics; 2009. p. 235–243.

[21] Li S, Huang CR, Zhou G, Lee SYM. Employing personal/impersonal views in supervised and semi-supervised sentiment classification. In: Proceedings of the 48th Annual Meeting of the Association for Computational Linguistics. Association for Computational Linguistics; 2010. p. 414–423.

[22] You Q, Luo J, Jin H, Yang J. Cross-modality consistent regression for joint visual-textual sentiment analysis of social multimedia. In: Proceedings of the Ninth ACM International Conference on Web Search and Data Mining. ACM; 2016. p. 13–22.

[23] Novak PK, Smailovic J, Sluban B, Mozetic I. Sentiment of emojis. PloS one. 2015;10(12):e0144296 10.1371/journal.pone.0144296 26641093 PMC4671607

[24] Ye Q, Zhang Z, Law R. Sentiment classification of online reviews to travel destinations by supervised machine learning approaches. Expert Systems with Applications. 2009;36(3):6527–6535. 10.1016/j.eswa.2008.07.035

[25] Yu H, Zhang C, Wang G. A tree-based incremental overlapping clustering method using the three-way decision theory. Knowledge-Based Systems. 2016;91:189–203. 10.1016/j.knosys.2015.05.028

[26] Zhou B. Multi-class decision-theoretic rough sets. International Journal of Approximate Reasoning. 2014;55(1):211–224. 10.1016/j.ijar.2013.04.006

[27] Pang B, Lee L. Seeing stars: Exploiting class relationships for sentiment categorization with respect to rating scales. In: Proceedings of the 43rd Annual Meeting on Association for Computational Linguistics. Association for Computational Linguistics; 2005. p. 115–124.

[28] Qu L, Ifrim G, Weikum G. The bag-of-opinions method for review rating prediction from sparse text patterns. In: Proceedings of the 23rd International Conference on Computational Linguistics. Association for Computational Linguistics; 2010. p. 913–921.

[29] Long C, Zhang J, Zhut X. A review selection approach for accurate feature rating estimation. In: Proceedings of the 23rd International Conference on Computational Linguistics: Posters. Association for Computational Linguistics; 2010. p. 766–774.

[30] Snyder B, Barzilay R. Multiple aspect ranking using the good grief algorithm. In: Proceedings of the 2007 Conference of the North American Chapter of the Association for Computational Linguistics: Human Language Technologies. Association for Computational Linguistics; 2007. p. 300–307.

[31] Wang H, Lu Y, Zhai C. Latent aspect rating analysis on review text data: a rating regression approach. In: Proceedings of the 16th ACM SIGKDD International Conference on Knowledge Discovery and Data Mining. ACM; 2010. p. 783–792.

[32] Cao L, Fei-Fei L. Spatially coherent latent topic model for concurrent segmentation and classification of objects and scenes. In: Proceedings of the 11th International Conference on Computer Vision. IEEE; 2007. p. 1–8.

[33] Ramage D, Hall D, Nallapati R, Manning CD. Labeled LDA: A supervised topic model for credit attribution in multi-labeled corpora. In: Proceedings of the 2009 Conference on Empirical Methods in Natural Language Processing. Association for Computational Linguistics; 2009. p. 248–256.

[34] Mei Q, Ling X, Wondra M, Su H, Zhai C. Topic sentiment mixture: Modeling facets and opinions in weblogs. In: Proceedings of the 16th International Conference on World Wide Web. ACM; 2007. p. 171–180.

[35] Li F, Huang M, Zhu X. Sentiment analysis with global topics and local dependency. In: Proceedings the 2010 International Conference of American association for Artificial Intelligence; 2010. p. 1371–1376.

[36] Lin C, He Y, Everson R, Ruger S. Weakly supervised joint sentiment-topic detection from text. IEEE Transactions on Knowledge and Data Engineering. 2012;24(6):1134–1145. 10.1109/TKDE.2011.48

[37] Forman G. An extensive empirical study of feature selection metrics for text classification. The Journal of Machine Learning Research. 2003;3:1289–1305.

[38] Liu X, Murata T. Advanced modularity-specialized label propagation algorithm for detecting communities in networks. Physica A: Statistical Mechanics and its Applications. 2010;389(7):1493–1500. 10.1016/j.physa.2009.12.019

[39] Brin S, Page L. The anatomy of a large-scale hypertextual web search engine. Computer Networks and ISDN Systems. 1998;30(1):107–117. 10.1016/S0169-7552(98)00110-X

[40] Austin D. How Google finds your needle in the web’s haystack. American Mathematical Society Feature Column. 2006;10:1–13.

[41] Lin CF, Wang SD. Training algorithms for fuzzy support vector machines with noisy data. Pattern Recognition Letters. 2004;25(14):1647–1656. 10.1016/j.patrec.2004.06.009

[42] Blitzer J, Dredze M, Pereira F. Biographies, bollywood, boom-boxes and blenders: Domain adaptation for sentiment classification In: ACL. vol. 7; 2007 p. 440–447.

[43] Tan S, Cheng X. Improving SCL model for sentiment-transfer learning. In: Proceedings of Human Language Technologies: The 2009 Annual Conference of the North American Chapter of the Association for Computational Linguistics, Companion Volume: Short Papers. Association for Computational Linguistics; 2009. p. 181–184.

[44] Wang H, Lu Y, Zhai C. Latent aspect rating analysis without aspect keyword supervision. In: Proceedings of the 17th ACM SIGKDD international conference on Knowledge discovery and data mining. ACM; 2011. p. 618–626.

[45] Wang S, Li D, Song X, Wei Y, Li H. A feature selection method based on improved fisher’s discriminant ratio for text sentiment classification. Expert Systems with Applications. 2011;38(7):8696–8702. 10.1016/j.eswa.2011.01.077

[46] Wang S, Li D, Zhao L, Zhang J. Sample cutting method for imbalanced text sentiment classification based on BRC. Knowledge-Based Systems. 2013;37:451–461. 10.1016/j.knosys.2012.09.003

[47] Shields MD, Teferra K, Hapij A, Daddazio RP. Refined stratified sampling for efficient Monte Carlo based uncertainty quantification. Reliability Engineering & System Safety. 2015;142:310–325. 10.1016/j.ress.2015.05.023

[48] Chang CC, Lin CJ. LIBSVM: A library for support vector machines. ACM Transactions on Intelligent Systems and Technology (TIST). 2011;2(3):1–39. 10.1145/1961189.1961199

[49] Baccianella S, Esuli A, Sebastiani F. SentiWordNet 3.0: An enhanced lexical resource for sentiment analysis and opinion mining. In: Proceedings of the 7th International Conference on Language Resources and Evaluation. vol. 10; 2010. p. 2200–2204.

